# The Relationship Between Lactate and Ventilatory Thresholds in Runners: Validity and Reliability of Exercise Test Performance Parameters

**DOI:** 10.3389/fphys.2018.01320

**Published:** 2018-09-25

**Authors:** Víctor Cerezuela-Espejo, Javier Courel-Ibáñez, Ricardo Morán-Navarro, Alejandro Martínez-Cava, Jesús G. Pallarés

**Affiliations:** Human Performance and Sports Science Laboratory, Faculty of Sport Sciences, University of Murcia, Murcia, Spain

**Keywords:** blood lactate, ventilation threshold, maximal aerobic speed, VO_2_max, endurance, maximal lactate steady state

## Abstract

The aims of this study were (1) to establish the best fit between ventilatory and lactate exercise performance parameters in running and (2) to explore novel alternatives to estimate the maximal aerobic speed (MAS) in well-trained runners. Twenty-two trained male athletes ($\dot{\text{V}}$O_2max_ 60.2 ± 4.3 ml·kg·min^−1^) completed three maximal graded exercise tests (GXT): (1) a preliminary GXT to determine individuals' MAS; (2) two experimental GXT individually adjusted by MAS to record the speed associated to the main **aerobic–anaerobic** transition events measured by indirect calorimetry and capillary blood lactate (CBL). Athletes also performed several 30 min constant running tests to determine the maximal lactate steady state (MLSS). Reliability analysis revealed low CV (<3.1%), low bias (<0.5 km·h^−1^), and high correlation (ICC > 0.91) for all determinations except V-Slope (ICC = 0.84). Validity analysis showed that LT, LT+1.0, and LT+3.0 mMol·L^−1^ were solid predictors of VT_1_ (−0.3 km·h^−1^; bias = 1.2; ICC = 0.90; *p* = 0.57), MLSS (−0.2 km·h^−1^; bias = 1.2; ICC = 0.84; *p* = 0.74), and VT_2_ (<0.1 km·h^−1^; bias = 1.3; ICC = 0.82; *p* = 0.9l9), respectively. MLSS was identified as a different physiological event and a midpoint between VT_1_ (bias = −2.0 km·h^−1^) and VT_2_ (bias = 2.3 km·h^−1^). MAS was accurately estimated (SEM ± 0.3 km·h^−1^) from peak velocity (V_peak_) attained during GXT with the equation: MAS_EST_ (km·h^−1^) = V_peak_ (km·h^−1^) ^*^ 0.8348 + 2.308. Current individualized GXT protocol based on individuals' MAS was solid to determine both maximal and submaximal physiological parameters. Lactate threshold tests can be a valid and reliable alternative to VT and MLSS to identify the workloads at the transition from aerobic to anaerobic metabolism in well-trained runners. In contrast with traditional assumption, the MLSS constituted a midpoint physiological event between VT_1_ and VT_2_ in runners. The V_peak_ stands out as a powerful predictor of MAS.

## Introduction

Numerous studies have embraced the question of how training programs based on individualized physiological parameters may increase cardiorespiratory performance in endurance sports like running or cycling. Evidence suggests establishing exercise workloads or intensities based on the individual physiological events (i.e., setting training zones) allows athletes to minimize injury and fatigue risks, but above all to enhance individual adaptations and respond to the training plan (Scharhag-Rosenberger et al., 2012; Mann et al., 2014; Wolpern et al., 2015). A recent review (Stöggl and Sperlich, 2015) addressed the fact that similar training intensity distribution shows different efficacy and adaptations depending on the competitive stage, the endurance discipline, and the athlete's performance levels. Thus, the more individualized and accurate the thresholds and training zones, the more precise the exercise prescription and the greater the athletes' adaptation and performance enhancement (García-Pallarés et al., 2009; Wolpern et al., 2015). From a competition point of view, exercise test performance parameters are useful to track cardiopulmonary and specific adaptations to the entire season training plan, and to explain performance (Lucía et al., 2000; Esteve-Lanao et al., 2007; García-Pallarés et al., 2009, 2010).

Physiological variables such as maximal oxygen uptake ($\dot{\text{V}}$O_2max_), submaximal metabolic inflection points like the pulmonary ventilation thresholds (VT) and lactate thresholds (LT), the maximal aerobic speed (MAS: the speed associated with $\dot{\text{V}}$O_2max_), or the peak velocity (V_peak_: the highest speed attained at the end of the test) are regular variables used by coaches and scientists to estimate and monitor running performance during training and competition events (Farrell et al., 1979; di Prampero et al., 1986; Stratton et al., 2009; McLaughlin et al., 2010). For the evaluation of these parameters in runners, it is common to use graded exercise tests (GXTs) on the treadmill, consisting of a series of stages lasting 1–5 min. Differences in the duration of each stage and the load increments can alter the cardiorespiratory and metabolic response, and therefore the measurement (Bentley et al., 2007; Julio et al., 2017). As suggested by pioneering studies (Buchfuhrer et al., 1983; Lukaski et al., 1989), recent investigations (Midgley et al., 2007) and reviews (Julio et al., 2017), traditional longer GXTs (i.e., 20–30 min) to determine LT including increments each 3–5 min would prevent the athlete from achieving their MAS due to accumulative fatigue, dehydration, muscle acidosis, and cardiovascular drift. This is critical because MAS is a pertinent and widespread criterion to set training intensities for endurance disciplines (Billat and Koralsztein, 1996; Jones and Carter, 2000). An interesting approach carried out with cyclists revealed that shorter protocols (12–14 min) including 1-min stages are valid both, to estimate submaximal metabolic inflection points (VT and LT), and to identify true values for $\dot{\text{V}}$O_2max_ and MAS in cyclists (Lucía et al., 1999, 2000; Gaskill et al., 2001; Midgley et al., 2007; Pallarés et al., 2016). However, the validity and reliability of GXT with 1-min stages protocol in runners needs to be fully verified.

Physiological response to exercise in endurance sports is commonly assessed though measurements based on ventilatory and lactate methods. However, the relationship between the two methods is not yet clear (Pallarés et al., 2016). Recent findings support the idea that a training model based on ventilatory thresholds (VT_1_ and VT_2_) could be very effective to set individual exercise intensity in endurance sports given that it takes into account individual metabolic responses (Wolpern et al., 2015). One of the most accurate systems to obtain these ventilatory responses is on the basis of gas exchange parameters using indirect calorimetry (Lucía et al., 2000; Gaskill et al., 2001; Pallarés et al., 2016). In VT_1_, the $\dot{\text{V}}$O_2_ and carbon dioxide production ($\dot{\text{V}}$CO_2_) increase proportionally, while HCO_3−_ acts to buffer lactic acid concentration in blood (Wasserman et al., 1973; Del Coso et al., 2009); this intensity is ideal for high-volume low-intensity exercise (Stöggl and Sperlich, 2014). In turn, in VT_2_, the blood lactate accumulation boosts and rises considerably and the system collapses due to the homeostatic compromise and metabolic acidosis (Wasserman et al., 1973; Jones et al., 2007); this intensity sets a critical limit for high-intensity interval training (Stöggl and Sperlich, 2014). However, gas exchange systems require the use of expensive equipment and laboratory conditions which most teams, coaches, and athletes are not equipped with or cannot afford.

A further method to set individual exercise intensity is based on capillary blood lactate (CBL) measurements (Beneke et al., 2011). A number of authors have defined a list of CBL parameters associated with specific exercise intensities such as LT (Wasserman et al., 1973), maximal lactate steady state (MLSS, Beneke and von Duvillard, 1996), OBLA (onset of blood lactate accumulation, Sjödin and Jacobs, 1981), or the D_MAX_ (Cheng et al., 1992)_._ An accurate detection of MLSS is particularly important due to it being considered the highest intensity in which glycogen stores are the main exercise limiting factor (Coyle et al., 1986) and constitutes a prominent part of aerobic training in world-class athletes (García-Pallarés et al., 2009, 2010). Although CBL methods are commonly used for coaches to set individual training workloads, the relationship between lactate-based parameters and VTs load intensities is still an open debate. In cyclists, it seems clear that workloads at the VT_1_ are very related to LT (Lucía et al., 1999; Amann et al., 2006; Pallarés et al., 2016). However, the estimation of VT_2_ from lactate methods generates some controversy. Traditionally, VT_2_ intensities have been associated with MLSS (Svedahl and MacIntosh, 2003). In contrast with this assumption, recent evidence in cyclists demonstrates that MLSS encompasses a different metabolic pathway and limiting factor than VT, and constitutes a midpoint between VT_1_ and VT_2_ (Pallarés et al., 2016; Peinado et al., 2016). The determination of VT_2_ is essential due to represents a turn point at which metabolic acidosis cannot be buffered by ventilation (Lucía et al., 2000) and sets a critical limit for high-intensity training (Stöggl and Sperlich, 2014). One previous study conducted with cyclists has attempted to clarify the relationship between VTs and CBL methods, and reported a high reliability and validity of the following relationships: (1) VT_1_ and LT, (2) VT_2_ and LT+2 mMol·L^−1^, and (3) MLSS and LT+0.5 mMol·L^−1^ (Pallarés et al., 2016). To the best of our knowledge, there are no previous studies examining these relationships in runners. This is an important gap considering the existing differences between cycling and running, such as the more impaired ventilation in cycling and the higher muscle mass involved, greater muscle pump efficiency, and the implication of eccentric muscle actions in running (Bijker et al., 2002; Millet et al., 2009). Given that these differences may alter the physiological response to exercise, prescribing training plans for runners based on cyclists' reference values could be imprecise. Thus, the relationships between CBL and VTs intensities in runners need to be fully clarified.

In addition to the aerobic–anaerobic transition, another ventilation parameter to predict running performance is the MAS (McLaughlin et al., 2010), considered as the minimum speed at which $\dot{\text{V}}$O_2max_ is reached (Lacour et al., 1991). As a rule of thumb, high intensity training in endurance athletes is established at 90–105% of the MAS (Stöggl and Sperlich, 2015). Given its importance for training plans and workload distribution, coaches and researchers have invested effort in designing maximal field tests to estimate the MAS and predict $\dot{\text{V}}$O_2max_ in endurance athletes to establish the aerobic performance limits (Léger and Boucher, 1980; Berthon et al., 1997). However, these tests have important limitations: (1) the equations proposed to estimate the MAS from field tests are not based on accurate measurements such as gas exchange systems using indirect calorimetry (Lucía et al., 2000; Gaskill et al., 2001), and (2) maximal efforts criteria were not tested to ensure reaching values of $\dot{\text{V}}$O_2max_ (ACSM, 2013). As stated above, a valid alternative to these field tests is to determine the MAS through GXT with 1-min increments using gas exchange systems. These short protocols allow the athletes to reach their maximal cardiac output, and therefore make possible obtaining a true V_peak_-value (Pallarés et al., 2016; Julio et al., 2017). Given that both MAS and V_peak_ correspond to very similar intensities (Lacour et al., 1991) the calculation of an estimated MAS (MAS_EST_) from the V_peak_, when gas exchange systems are not available, seems promising. However, this hypothesis is still to be proven.

Therefore, the aims of this study were (1) to establish the best fit between ventilatory and lactate exercise performance parameters in running and (2) to explore novel alternatives to estimate essential running performance indicators such as the MAS from similar intensity parameters like the V_peak_ when gas exchange systems are not available.

## Methods

### Participants

Twenty-two trained male athletes (runners and triathletes) volunteered to participate in this study (age 25.9 ± 8.0 years, body mass 68.2 ± 6.1 kg, height 174.8 ± 5.8 cm, body fat 11.4 ± 1.9%, $\dot{\text{V}}$O_2max_ 60.2 ± 4.3 ml·kg·min^−1^, endurance training experience 7.1 ± 4.0 years). All participants were competing at regional and national level races and following a regular training load of 4–6 days per week, 1–2 h per day. Measurements were obtained during the pre-competitive season. All participants underwent a complete medical examination (including ECG) that showed all were in good health. No physical limitations or musculoskeletal injuries that could affect testing procedures were reported. None of the subjects were taking drugs, medications, or dietary supplements known to influence physical performance. The Bioethics Commission of the University of Murcia approved the study, which was carried out according to the declaration of Helsinki. Subjects were verbally informed about the experimental procedures and possible risk and benefits. Written informed consent was obtained from all subjects.

### Experimental design

Participants visited the lab 5–7 times separated by 2–7 days. All participants had at least 6 months of familiarization with the testing procedures used in this investigation. On the first day, participants completed a preliminary GXT with 1-min increments (GXT_PRE_) to determine individuals' MAS and V_peak_, including 48–72 h rest before the next session. In the following two sessions, separated by 48 h, athletes performed two identical experimental GXT (GXT_EXP_ 1 and GXT_EXP_ 2). For these two GXT_EXP_ protocols, initial running speed and workload increments were individually set according to participants' V_peak_ previously determined in the GXT_PRE_.

The GXT_EXP_ started with a 5-min warm-up at 13 km·h^−1^ less than each athlete's V_peak_ followed, without a break, by a GXT 1-min (i.e., increments of 1 km·h^−1^·min^−1^) until exhaustion. Lastly, athletes came back to the lab two to three more times to perform a 30 min submaximal constant running test to determine the speed associated with the MLSS (Beneke, 2003). To maintain physical performance during the investigation period (2–3 weeks) participants followed an individual training protocol consisting in: running sessions (runners) or swimming, cycling, and running sessions (triathletes) of 90 min at individual VT_1_ intensity interspersed with efforts of 5–7 min at 90–95% of VT_2_ intensity each 20 min. Training sessions were repeated each 48 h with 24 h rest before each evaluation to ensure a full recovery.

### Individualized maximal treadmill GXT protocol

All the running trials were performed on the same treadmill (HP Cosmos Pulsar, HP Cosmos Sports and Medical GMBH, Nussdorf Traunstein, Germany) with an incline of 1.0% (Jones and Doust, 1996). Evaluations were performed under similar environmental conditions (21–24°C and 45–55% relative humidity) at the same time of day (16:00 to 19:00 h) to minimize the circadian rhythm effects (Mora-Rodríguez et al., 2015). Air ventilation was controlled with a fan positioned 1.5 m from the subject's chest at a wind velocity of 2.55 m·s^−1^.

The GXT_PRE_ under medical supervision to fulfill three objectives: (1) discard cardiovascular diseases, (2) to minimize the bias of progressive learning on test reliability, and (3) to determine the athletes' MAS and V_peak_ subsequently used to set up the individualized GXT_EXP_ workload (i.e., treadmill speed). Participants' HR was monitored by standard 12 lead ECG (Quark T12, Cosmed, Italy), ventilatory performance ($\dot{\text{V}}$O_2_, $\dot{\text{V}}$O_2max_, and VE) was recorded on a breath-by-breath basis using a metabolic cart averaging data every 5 s (MetaLyzer 3B-R3, Cortex Biophysik GmbH, Leipzig, Germany) and the rate of perceived exertion (RPE) was assessed using the 6–20 Borg Scale (Borg, 1998) every 2 min. The MAS was determined from metabolic cart measurements as the first running velocity where $\dot{\text{V}}$O_2max_ was reached (Billat and Koralsztein, 1996). The V_peak_ was automatically obtained from the treadmill software using the Kuipers et al.'s formula (Kuipers et al., 2003): V_peak_ = V_complete_ + (*Inc* ^*^ *t*/*T*), in which V_complete_ is the running velocity of the last complete stage, *Inc* is the speed increment (i.e., 1 km·h^−1^), *t* is the number of seconds sustained during the incomplete stage and *T* is the number of seconds required to complete a stage (i.e., 60 s).

The two GTX_EXP_ were individually set up according to the MAS previously determined in the preliminary test (GXT_PRE_), as follows: starting with a 5-min warm-up at 13 km·h^−1^ less than each athlete's V_peak_, followed, without a break, by a GXT 1-min (i.e., increments of 1 km·h^−1^·min^−1^) until exhaustion. Ventilatory parameters and RPE were assessed as aforementioned in the GXT_PRE_. The HR was continuously monitored (V800, Polar, Finland). Capillary blood lactate samples from the finger were collected (Lactate Pro, Arkray, Japan) every 2 min (i.e., each 2 km·h^−1^ increments). The design of this particular protocol and its duration (min–max) were deliberate, given that: (1) It allows a clear detection of ventilatory thresholds (VT_1_ and VT_2_) by indirect calorimetry (Lucía et al., 1999, 2000; Pallarés et al., 2016); (2) It is effective in determining a true $\dot{\text{V}}$O_2max_ (Midgley et al., 2007); (3) The protocol duration was short enough (12–14 min) to avoid the local acidosis and HR rise (cardiac drift) to obtain a true maximum cardiovascular performance (Dawson et al., 2005); (4) The short duration allows athletes to achieve a true MAS and V_peak_ (Julio et al., 2017); and (5) By the end of the test, seven to nine capillary blood samples can be collected from each participant before exhaustion, which enable the plotting of a complete lactate curve. In particular, fingerprint blood samples were collected by a specialist placed beside the treadmill without any pause during the participants' running test (i.e., in movement) to make the process less invasive and ensure a constant effort during the GXT protocols.

Maximal effort criteria (ACSM, 2013) were considered to verify the outcomes, from which participants must reach at least three from the list: (i) failure of HR to increase with further increases in exercise intensity; (ii) a plateau in $\dot{\text{V}}$O_2_ (or failure to increase $\dot{\text{V}}$O_2_ by 150 mL·min^−1^) with increased workload; (iii) a respiratory exchange ratio (RER) ≥1.10; CBL >8 mmol·L^−1^; (iv) a rating of perceived exertion (RPE) >17 on the 6–20 scale. If verified, physiological parameters were determined, and the individuals' treadmill speed at each of the physiological parameters studied were considered for subsequent analysis. Blood lactate analyzer and indirect calorimetry devices were calibrated before each test according to the manufacturer's instructions.

### Determination of MLSS

Several 30 min constant workloads on a treadmill were performed to identify the highest workload (km·h^–1^) at which CBL increased >1 mMol·L^–1^ between the 10th and 30th min of exercise (Beneke, 2003). After 7 days from the second GXT_EXP_, all participants performed the first MLSS trial at the individual workload associated to their 85% of VT_2_, based on previous studies (Llodio et al., 2016; Pallarés et al., 2016). Depending on the results of the first MLSS**-**test, successive trials with a 48-h rest between sessions were increased or decreased 0.5 km·h^–1^ until MLSS criteria was fulfilled (Pallarés et al., 2016).

### Determination of ventilation parameters

**Figure 1:**
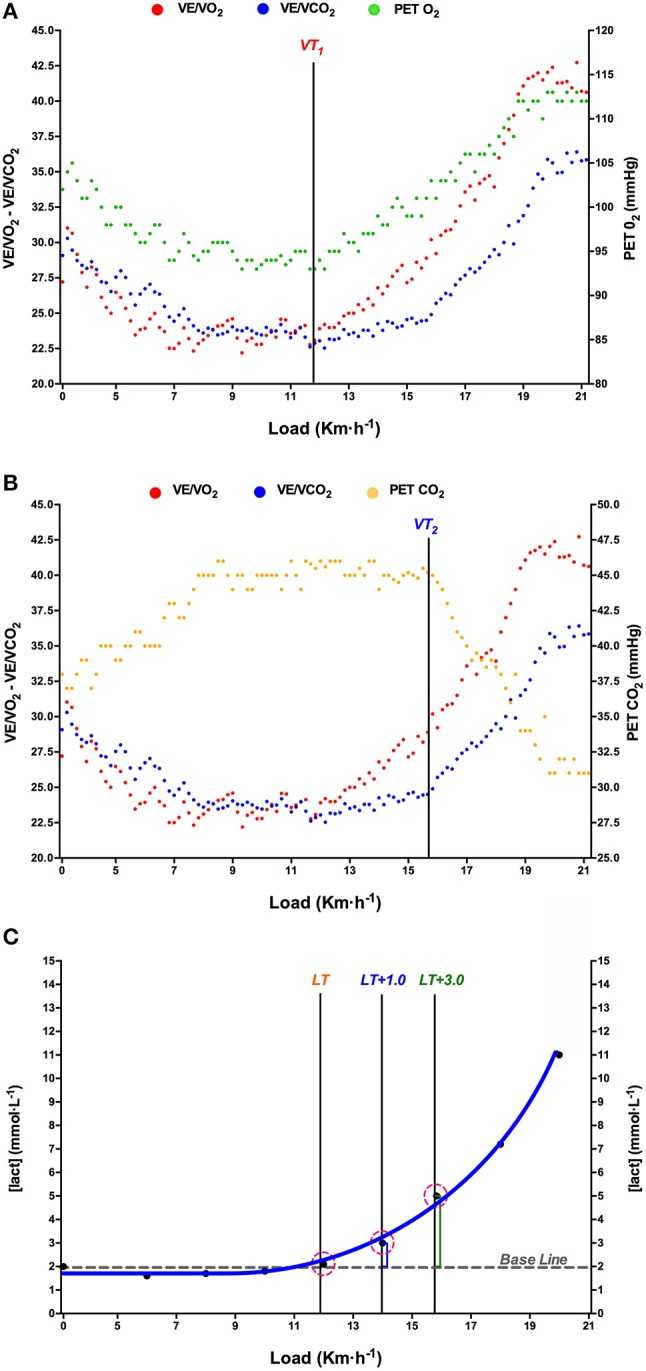
Example of determination of ventilatory thresholds (VT1, **A**; VT2, **B**), and lactate threshold (LT, **C**) in one test. Each gas-exchange data point corresponds to a 5-s interval. $\dot{\text{V}}$E/$\dot{\text{V}}$O_2_, ventilatory equivalent for oxygen; $\dot{\text{V}}$E/$\dot{\text{V}}$CO_2_, ventilatory equivalent for carbon dioxide; P_ET_CO_2_, end-tidal pressure of oxygen; end-tidal pressure of carbon dioxide (P_ET_CO_2_).

VT_1_ was determined using the criteria of an increase in both ventilatory equivalent of oxygen ($\dot{\text{V}}$_E_/$\dot{\text{V}}$O_2_) and end-tidal pressure of oxygen (P_ET_O_2_) with no concomitant increase in ventilatory equivalent of carbon dioxide ($\dot{\text{V}}$_E_/$\dot{\text{V}}$CO_2_). VT_2_ was determined using the criteria of an increase in both the $\dot{\text{V}}$_E_/$\dot{\text{V}}$O_2_ and $\dot{\text{V}}$_E_/$\dot{\text{V}}$CO_2_ and a decrease in P_ET_CO_2_ (Lucía et al., 2000; Figures 1A,B). V-Slope load was identified in that intensity of exercise which, in a plot of the minute production of CO_2_ over the minute utilization of oxygen ($\dot{\text{V}}$O_2_), shows an increase in the slope above 1.0 (Wasserman et al., 1973; Gaskill et al., 2001). The $\dot{\text{V}}$O_2max_ was defined as the highest plateau (two successive maximal within 150 mL·min^−1^, averaging the data every 5 s) reached. MAS was defined as the minimum speed at which maximum oxygen uptake $\dot{\text{V}}$O_2max_ is reached (Lacour et al., 1991). V_peak_ was taken from the highest velocity reached during this GXT protocol and calculated according to the Kuipers et al. (2003).

### Determination of lactate parameters

LT was determined by examining the CBL speed relationship ([Lact]_blood_/ km·h^−1^) during the GXT as the highest speed not associated with a rise in CBL above baseline (Weltman et al., 1990). Baseline CBL was the average during the initial stages with values 0.8 mMol·L^−1^ above rest state. This always occurred just before the curvilinear increase in blood lactate observed at subsequent exercise intensities (Coyle et al., 1983; Lucía et al., 2000). Lactate Threshold + 1.0 mMol·L^−1^ (LT+1.0) represents the speed which causes an increase of 1 mMol·L^−1^ above baseline measurements (Coyle et al., 1983). Following this criterion, five LT-based events were established as previously described (Pallarés et al., 2016): LT+0.5, LT+1, LT+1.5, LT+2.0, LT+2.5, and LT+3.0 mMol·L^−1^. D_MAX_ method was determined by plotting the lactate response to exercise intensity in a third-order polynomial regression curve. D_MAX_ was defined as the point on the regression curve that yields the maximal distance to the straight line formed by the two end points of the curve (Cheng et al., 1992). Onset of blood lactate accumulation (OBLA_4mM_) was defined as the exercise intensity identified by interpolation across the 4 mMol·L^−1^ point in the plot of [Lact]_blood_ during incremental exercise (Sjödin and Jacobs, 1981). Two independent observers detected all ventilatory and LT following the criteria previously described. If they did not agree, the opinion of a third investigator was sought (Lucía et al., 1999; Figure 1C).

### Statistical analyses

Standard statistical methods were used for the calculation of means, standard deviations (SD), and 95% confidence interval. The reliability of ventilation and lactate parameters was analyzed comparing the consistence among trials (i.e., GXT_EXP_ 1 vs. GXT_EXP_ 2) by calculating the coefficient of variation (CV), intraclass correlation coefficient (ICC), and Bland–Altman plots. Linear regression analysis was employed to estimate a theoretical MAS (MAS_EST_) from the average of the V_peak_ achieved at the end of the two GXT_EXP_ trials. Validity analysis of the ventilatory thresholds (VT_1_ and VT_2_), MLSS, and MAS against the other parameters was conducted over the means obtained in the trials by ANOVA, ICC, and Bland-Altman bias. Analyses were performed using GraphPad Prism 6.0 (GraphPad Software, Inc., CA, USA) and SPSS software version 19.0 (IBM Corp., Armonk, NY, USA).

## Results

**Figure 2:**
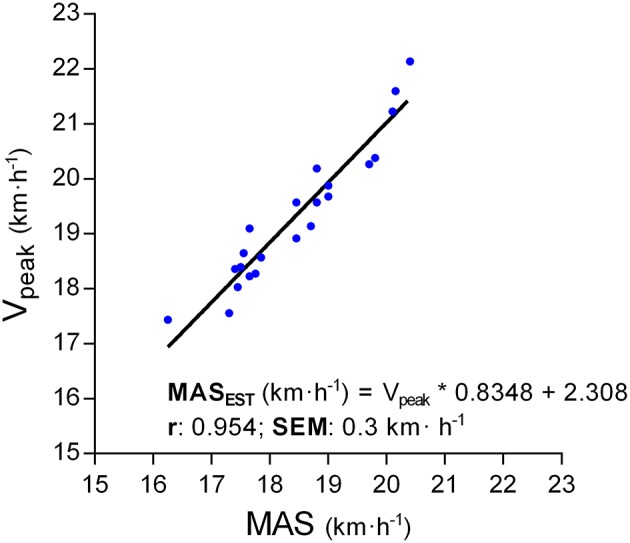
Linear regression estimating a theoretical maximal aerobic speed (MAS_EST_) from the average of the fastest velocity achieved at the end of the GXT_EXP_ trials (V_peak_).

All participants reached at least two of the criteria for achievement of maximal efforts during all the GXT-tests, therefore maximal ventilation and cardiovascular performance was verified. The initial speed ranged from 6 to 10 km·h^−1^ and no fatigue was detected following the warm-up (i.e., all participants maintained a RER < 0.85 and CBL under the baseline). The V_peak_ reached during the GXT_PRE_ ranged from 18 to 22 km·h^−1^. Linear regression analysis (Figure 2) revealed a very strong association between MAS and V_peak_ (*p* < 0.01; r = 0.954; SEM = 0.3 km·h^−1^) and yielded the equation:

$$\begin{array}{l} MAS_{EST}\;\left(km\cdot h^{-1}\right)\;=\;V_{peak}\;\left(km\cdot h^{-1}\right){\;}^{*}0.8348\;+\;2.308 \end{array}$$

Intra-subject reliability between GXT_EXP_ trials (Table 1) revealed low CV (<3.1%), low bias (<0.5 km·h^−1^), and high correlation (ICC > 0.91) for all determinations except V-Slope (ICC = 0.84).

**Table 1:** Reliability of lactate and ventilatory tests.

	**CV**	**ICC**	**Bland altman**	
	**%**	**r**	**Bias (SD)**	***LoA 95%***
VT_1_	2.08	0.98	0.22 (0.47)	−0.71; 1.15
VT_2_	1.92	0.95	0.13 (0.63)	−1.11; 1.37
MAS	2.20	0.91	0.36 (0.64)	−0.9; 1.62
LT	1.99	0.98	0.09 (0.43)	−0.76; 0.94
LT+0.5	1.23	0.96	0.07 (0.87)	−1.64; 1.78
LT+1.0	3.49	0.96	0.07 (0.79)	−1.48; 1.62
LT+1.5	3.08	0.96	0.10 (0.76)	−1.39; 1.59
LT+2.0	2.99	0.97	0.07 (0.69)	−1.29; 1.43
LT+2.5	2.53	0.96	0.11 (0.73)	−1.33; 1.55
LT+3.0	2.46	0.96	0.10 (0.77)	−1.41; 1.61
V-Slope	2.58	0.84	0.31 (1.11)	−1.87; 2.49
D_MAX_	2.12	0.94	0.27 (1.09)	−1.87; 2.41
OBLA_4mM_	3.08	0.96	0.48 (0.97)	−1.43; 2.39
V_peak_	2.79	0.94	0.12 (0.42)	−0.71; 0.95

*CV, Coefficient of variation; ICC, Intraclass coefficient; and Bland-Altman results. V_peak_, the fastest velocity achieved at the end of the graded exercise testing protocol; MAS, Maximal aerobic speed; VT_1_, First ventilatory threshold; MLSS, Maximal lactate steady state; VT_2_ Second, ventilatory threshold; LT, Lactate threshold; LT+0.5,+1.0,+1.5,+2.0,+2.5,+3.0, Concentrations above lactate threshold; D_MAX_, Maximum distance between the slope of a polynomial and the line connecting both ends; OBLA_4mMol_, Onset blood lactate accumulation 4 mM; LoA, 95% limit of agreement*.

Table 2 shows the validity analysis comparing VT_1_, MLSS, VT_2_, and MAS workloads against the rest of the parameters. The strongest associations (ICC > 0.82, *p* > 0.57) were: VT_1_ with LT (−0.3 ± 1.2 km·h^−1^), MLSS with LT+1.0 (−0.2 ± 1.2 km·h^−1^), VT_2_ with LT+3.0 (<0.1 ± 1.3 km·h^−1^), and MAS with MAS_EST_ (<0.1 ± 0.4 km·h^−1^).

**Table 2:** Validity results of used methods.

	**Speed (km·h^−1^)**	**Blood lactate (mMol·L^−1^)**	**VT**_**1**_				**MLSS**				**VT**_**2**_				**MAS**			
			**Speed: 11.5** ± **1.8 km**·**h**^**−1**^				**Speed: 13.5** ± **1.1 km**·**h**^**−1**^				**Speed: 15.8** ± **1.4 km**·**h**^**−1**^				**Speed: 18.1** ± **1.3 km**·**h**^**−1**^			
	**M ± SD**	**M ± SD**	**Student *t***	**ICC**	**Bland altman**		**Student *t***	**ICC**	**Bland altman**		**Student *t***	**ICC**	**Bland altman**		**Student *t***	**ICC**	**Bland altman**	
			***p***		***Bias (SD)***	***LoA 95%***	***p***		***Bias (SD)***	***LoA 95%***	***p***		***Bias (SD)***	***LoA 95%***	***p***		***Bias (SD)***	***LoA 95%***
VT_1_	11.5 ± 1.8	2.2 ± 0.7																
MLSS	13.5 ± 1.1	3.3 ± 1.1	<0.01	0.91	−2.0 (0.9)	−3.8; −0.3												
VT_2_	15.8 ± 1.4	5.7 ± 1.9	<0.01	0.92	−4.3 (0.9)	−6.1; −2.6	<0.01	0.94	−2.3 (0.7)	−3.6; −1.0								
MAS	18.1 ± 1.3	10.6 ± 3.1	<0.01	0.86	−7.6 (2.0)	−11.6; −3.7	<0.01	0.95	−5.0 (0.6)	−6.2; −3.9	<0.01	0.91	−3.1 (1.8)	−6.7; 0.5				
LT	11.8 ± 1.8	2.3 ± 0.4	**0.57**	**0.90**	–**0.3 (1.2)**	–**2.7; 2.1**	<0.01	0.82	1.8 (1.3)	−0.9; 4.4	<0.01	0.83	4.1 (1.4)	1.4; 6.9	<0.01	0.75	6.7(1.5)	3.8; 9.7
LT+0.5	12.7 ± 2.1	2.8 ± 0.4	0.03	0.93	−1.3 (1.1)	−3.5; 0.9	0.15	0.82	0.7 (1.4)	−2.0; 3.5	<0.01	0.84	3.0 (1.4)	0.3; 5.8	<0.01	0.76	5.7 (1.6)	2.7; 8.8
LT+1.0	13.6 ± 2.0	3.3 ± 0.4	<0.01	0.93	−2.2 (1.1)	−4.4; −0.1	**0.74**	**0.84**	–**0.2 (1.2)**	–**2.6; 2.3**	<0.01	0.84	2.1 (1.4)	−0.7; 4.9	<0.01	0.77	4.8 (1.5)	2.0; 7.7
LT+1.5	14.3 ± 1.9	3.8 ± 0.4	<0.01	0.93	−2.8 (1.1)	−5.0; −0.7	0.09	0.85	−0.8 (1.2)	−3.2; 1.6	<0.01	0.84	1.5 (1.3)	−1.1; 4.1	<0.01	0.78	4.1 (1.4)	1.4; 7.0
LT+2.0	14.8 ± 1.9	4.3 ± 0.4	<0.01	0.92	−3.4 (1.1)	−5.6; −1.3	0.01	0.85	−1.3 (1.4)	−4.0; 1.4	0.07	0.84	0.9 (1.3)	−1.7; 3.5	<0.01	0.78	3.6 (1.4)	0.9; 6.4
LT+2.5	15.3 ± 1.9	4.8 ± 0.4	<0.01	0.91	−3.9 (1.1)	−6.1; −1.8	<0.01	0.85	−1.8 (1.1)	−4.1; 0.5	0.37	0.83	0.4 (1.3)	−2.4; 3.2	<0.01	0.78	3.1 (1.4)	0.4; 5.9
LT+3.0	15.8 ± 1.8	5.3 ± 0.4	<0.01	0.91	−4.3 (1.1)	−6.5; −0.3	<0.01	0.85	−2.3 (1.2)	−4.6; −0.1	**0.99**	**0.82**	<**0.1 (1.3)**	–**2.5; 2.6**	<0.01	0.78	2.7 (1.4)	−0.1; 5.4
V-Slope	14.6 ± 1.4	4.4 ± 2.1	<0.01	0.76	−3.1 (1.5)	−6.1; −0.2	0.01	0.85	−1.1 (1.2)	−3.6; 1.4	0.01	0.73	1.2 (1.4)	−1.6; 4.0	<0.01	0.57	3.9 (1.5)	1.0; 6.8
D_MAX_	14.1 ± 1.6	3.7 ± 1.0	<0.01	0.96	−2.6 (0.8)	−4.2; −1.1	0.19	0.94	−0.6 (0.8)	−2.1; 0.9	<0.01	0.91	1.7 (1.0)	−0.3; 3.7	<0.01	0.92	4.4 (0.9)	2.6; 6.2
OBLA_4mM_	14.5 ± 2.1	4.0 ± <0.1	<0.01	0.93	−3.0 (1.1)	−5.2; −0.9	0.06	0.84	−1.0 (1.3)	−3.6; 1.6	0.02	0.86	1.3 (1.4)	−0.9; 3.5	<0.01	0.78	3.9 (1.5)	1.0; 7.0
V_peak_	19.3 ± 1.3	12.0 ± 3.9	<0.01	0.93	−8.5 (2.1)	−12.7; −4.4	<0.01	0.96	−5.8 (0.6)	−7.0; −4.7	<0.01	0.93	−3.9 (2.0)	−7.9; 0.1	0.02	0.97	−0.9 (0.5)	−1.9; 0.2
MAS_EST_	18.4 ± 1.1	–	<0.01	0.88	−7.6 (2.0)	−11.6; −3.7	<0.01	0.96	−4.9 (0.5)	−6.0; −4.0	<0.01	0.91	−3.1 (1.9)	−6.9; 0.7	>**0.99**	**0.98**	<**0.1 (0.4)**	–**0.9; 0.9**

*Comparison of running speeds at VT_1_, MLSS, VT_2_, and MAS against the rest of parameters.VT_1_, First ventilatory threshold; MLSS, Maximal lactate steady state; VT_2_ Secondary ventilatory threshold; MAS, Maximal aerobic speed; LT, Lactate threshold; LT+0.5,+1.0,+1.5,+2.0,+2.5,+3.0, Concentrations above lactate threshold; D_MAX_, Maximum distance between the slope of a polynomial and the line connecting both ends; OBLA_4mMol_, Onset blood lactate accumulation 4 mMol·L^−1^; V_peak_, Fastest velocity achieved at the end of the graded exercise testing protocol; MAS_EST_, Theoretical MAS estimated from V_peak_. The most powerful relationships are highlighted in bold. LoA, 95% limit of agreement*.

Table 3 shows the 95% confidence interval for main physiological parameters under study.

**Table 3:** 95% confidence interval values for main physiological events.

	**MAS (%)**	**HR_Max_ (%)**	**HRR (%)**	**RPE_6−20_**
VT_1_	59–65	77–81	68–74	10–12
MLSS	72–74	85–87	79–83	12–13
VT_2_	84–87	91–93	81–98	15–16
MAS	100	98–100	98–100	18–20

*VT_1_, First ventilatory threshold; MLSS, Maximal lactate steady state; VT_2_ Second, ventilatory threshold; MAS, Maximal aerobic speed; HR_Max_, Maximal heart rate; HRR, Heart rate reserve; RPE, Rate of perceived exertion*.

## Discussion

The main findings of the current study were that (1) LT obtained during a 12–14 min, 1 km·h^−1^ per minute GXT is a valid method to determine the main physiological parameters of the aerobic–anaerobic transition, (2) LT, LT+1 and LT+3.0 are solid predictors of VT_1_, MLSS, and VT_2_, respectively, (3) the MLSS was identified as a midpoint between VT_1_ and VT_2_, and (4) an estimated maximal aerobic speed (MAS_EST_) can be accurately obtained (error ± 0.3 km·h^−1^) from the fastest speed achieved during the current GXT (V_peak_). This study adds to the existing literature by providing a valid alternative test based on blood lactate to obtain performance workloads without the need of using indirect calorimetry (less affordable technology). In addition, we contribute with an accurate method to estimate the MAS, which is one of the most used indicators to set training intensities in running. To our knowledge, this is the first report examining the validity and reliability of such an extensive battery of tests and parameters to determine critical workloads in runners.

The high reliability values found in physiological measurements between the two GXT_EXP_ treadmill trials concurs with those previously reported in cycle ergometer (Pallarés et al., 2016). In addition, our results allow us to discourage using V-Slope when other parameters are available. Although the causes that might explain these effects are very difficult to isolate and quantify, it is arguable that an individualized workload adjustment approach accounted for these increments (García-Pallarés et al., 2009; Wolpern et al., 2015). In the current GXT with 1-min increments, athletes started at 13 km·h^−1^ below their V_peak_, previously determined during the GXT_PRE_ session. By doing this, it is guaranteed that the athlete is running at the optimal intensity to end up at their maximum workload after 12–14 min avoiding cardiac drift, local acidosis, and allowing a clear detection of ventilatory and LT, additionally getting maximal values of $\dot{\text{V}}$O_2max_ Considering this information, individual GXT protocols based on athletes' maximal speed should be developed to enhance the consistency of data during physiological evaluations.

A number of studies have investigated the relationship between ventilatory threshold and blood lactate concentration in endurance athletes. Authors agreed that workloads at the first ventilatory threshold (i.e., VT_1_) are strongly related to the workload at which lactate starts to increase above resting values (LT; Wasserman et al., 1973; Lucía et al., 2000; Pallarés et al., 2016). Our findings corroborate this association between VT_1_ and LT but showing a greater external workload in runners (VT_1_ = 59–65% of MAS) compared to cyclists [VT_1_ ~ 51.5% of maximal aerobic power (MAP)]. These findings suggest that running describes a great relative external workload associated with the VT_1_ response. Therefore, smaller errors in detecting ventilatory thresholds may have a greater negative impact on the running performance compared to other disciplines like cycling, for instance, misguided training prescription, undesirable physical adaptations, and a greater probability of the appearance of the interference phenomenon during concurrent training (García-Pallarés and Izquierdo, 2011). In turn, there is no clear agreement about which LT better reflects VT_2_ intensities. A previous experiment in cyclists (Pallarés et al., 2016) determined a high correlation between VT_2_ and LT+2 mMol·L^−1^, followed by the OBLA_4mM_, which established high intensities at ~80% of their maximal aerobic power (MAP). Interestingly enough, we identified a greater CBL in runners during the transition phase, locating the VT_2_ at LT+3 mMol·L^−1^ intensities, setting the high intensity limit at 84–87% of the MAS. The existing physiological differences between running and cyclists may explain these disparities. Millet et al. (2009) reviewed the literature and identified a list of potential distinguishing factors between running and cycling physiological demands. The authors pointed out differences on ventilatory responses to exercise in terms of exercise-induced arterial hypoxaemia, O_2_ diffusion capacity, ventilatory fatigue, and pulmonary mechanics. Moreover, other factors like running/cycling economy (higher delta efficiency in running), muscle recruitment patterns (greater muscle mass involved and eccentric phase activity in running), and ventilation impairment (higher in cycling) may account for these differences.

The MLSS constitutes another essential physiological event in endurance performance, as it is the maximal workload that can be maintained without elevations in blood lactate concentration (MLSS). Previous authors have proposed that MLSS workload coincides with the one for VT_2_ (Smekal et al., 2012). In contrast to this assumption, recent investigations in cyclists elucidated that MLSS may correspond to a lower exercise intensity of VT_2_ and matches better with the midpoint between both ventilatory thresholds (Pallarés et al., 2016; Peinado et al., 2016). In support of this theory, our findings revealed that MLSS intensity (72–74% of MAS) constitutes a transition between VT_1_ (59–65% of MAS) and VT_2_ (84–87% of MAS). Moreover, MLSS was highly associated with LT+1 mMol·L^−1^. In cyclists, MLSS has been associated with LT+0.5 mMol·L^−1^ (Pallarés et al., 2016). These increments on CBL at the same relative intensity might indicate a greater energy cost in running at MLSS workload, which may imply earlier fatigue and lower performance by accelerating glycogen depletion (Coyle et al., 1986).

A main contribution of the current study is to provide an estimated MAS (MAS_EST_) from the maximal speed achieved (V_peak_) at the end of the GXT protocol with a minimal error of ±0.3 km·h^−1^. Main physiological events (VT and MLSS) are related to a given percentage of MAS (Pallarés et al., 2016), therefore the estimation of MAS would allow coaches to determine effective working ranges (Table 3) and training zones (Table 4) with an error of <0.5%. Although there are other track tests to estimate the MAS (e.g., Léger and Boucher, 1980), the current protocol adds to the existing methods the possibility to design individualized training routines based on athletes' MAS without the need of indirect calorimetry o CBL records. It is important to mention that, given the originality of the proposal, the current outcomes have been shown to be valid only for the subject group that was tested. Future research should extend these findings to examine the validity of the MAS_EST_ compared to estimations from existing field tests.

**Table 4:** Personal author's approach for exercise prescription (training zones).

**Intensity**	**Zone**	**MAS (%)**	**V_peak_ (%)**	**HR_Max_ (%)**	**HRR (%)**	**RPE_6−20_**
70–90% VT_1_ or LT	R0	43–56	40–52	55–70	50–64	8–10
90–110% VT_1_ or LT	R1	57–68	53–64	71–83	65–77	11–12
95–105% MLSS or LT+1.0	R2	69–79	65–75	84–88	78–84	13–14
95–105% VT_2_ or LT+3.0	R3	80–93	76–89	89–94	85–93	15–16
95–105% $\dot{\text{V}}$O_2_max	R3+	94–105	90–100	>95	>94	>17

*V_peak_, the fastest velocity achieved at the end of the graded exercise testing protocol; VT_1_, First ventilatory threshold; MLSS, Maximal lactate steady state; VT_2_ Second, ventilatory threshold; MAS, Maximal aerobic speed; HR_Max_, Maximal heart rate; HRR, Heart rate reserve; RPE, Rate of perceived exertion*.

It is noteworthy that, given the high inter-individual response of training adaptations in endurance exercise (Bouchard et al., 1986), a similar workload distribution may not have the same effect among athletes, even if they are from the same discipline and compete at high level (Stöggl and Sperlich, 2015). In this case, an individual assessment is required to detect specific workloads. However, this implies indirect calorimeters methods which are expensive and out of reach for the majority of coaches and athletes. In this study, we provide a valid and reliable alternative to estimate critical workloads (VT_1_, MLSS, and VT_2_) using a cheaper and affordable method such as CBL. Furthermore, the current GXT individualized protocol (i.e., starting at 13 km·h^–1^ below the athlete's MAS with increments of 1 km·h^–1^/min until) appears to be a promising method to determine training zones in well-trained runners. What is now required is to test the effectiveness of training plans according to the current 5-zone proposal (Table 4). In addition, future investigations should examine the validity of this protocol in amateur and female runners to enhance its applicability within the endurance sport community.

## Ethics statement

All procedures performed in this study involving human participants were in accordance with the ethical standards of the institutional Human Research Ethics Committee and with the 1964 Helsinki declaration and its later amendments or comparable ethical standards. The study was approved by the Bioethics Commission of the University of Murcia. Written informed consent was obtained from all subjects prior to participation.

## Author contributions

JP and VC-E: Conception and design of the experiments; JP, VC-E, RM-N, and AM-C: Pre-testing, experimental preparation, and data collection; VC-E, JC-I, and JP: data analysis. The first draft of the manuscript was written by VC-E, JC-I, and JP. All co-authors edited and proofread the manuscript and approved the final version.

### Conflict of interest statement

The authors declare that the research was conducted in the absence of any commercial or financial relationships that could be construed as a potential conflict of interest.
